# Statin use and lung cancer risk in chronic obstructive pulmonary disease patients: a population-based cohort study

**DOI:** 10.1186/s12931-020-01344-w

**Published:** 2020-05-19

**Authors:** AJN Raymakers, D. D. Sin, M. Sadatsafavi, JM FitzGerald, C. A. Marra, L. D. Lynd

**Affiliations:** 1 Collaboration for Outcomes Research and Evaluation (CORE), Faculty of Pharmaceutical Sciences, University of British Columbia, 2405 Wesbrook Mall, Vancouver, British Columbia V6T1Z3 Canada; 2 BC Cancer, Vancouver, Canada; 3 Faculty of Health Sciences, Simon Fraser University, Burnaby, Canada; 4 Centre for Heart Lung Innovation, St Paul’s Hospital, Vancouver, Canada; 5 Division of Respiratory Medicine, Faculty of Medicine, University of British Columbia, Vancouver, Canada; 6 School of Pharmacy, University of Otago, Dunedin, New Zealand; 7 Centre for Health Evaluation and Outcome Sciences, Providence Health Research Institute, Vancouver, Canada

**Keywords:** Statins, Pharmacoepidemiology, Health services research, Administrative data, Epidemiology, Real-world data

## Abstract

**Background:**

Patients living with chronic obstructive pulmonary disease (COPD) are at an increased risk of lung cancer. A common comorbidity of COPD is cardiovascular disease; as such, COPD patients often receive statins. This study sought to understand the association between statin exposure and lung cancer risk in a population-based cohort of COPD patients.

**Methods:**

We identified a population-based cohort of COPD patients based on having filled at least three prescriptions for an anticholinergic or short-acting beta-agonist (SABA). We used an array of methods of defining medication exposure including three conventional methods (ever statin exposure, cumulative duration of use, and cumulative dose) and two novel methods (recency-weighted cumulative duration of use and recency-weighted cumulative dose). To assess residual confounding, a negative control exposure was used to test the validity of our results. All exposure variables were time-dependent.

**Results:**

The population-based cohort of COPD had 39,879 patients with mean age of 70.6 (SD: 11.2) years and, of which, 53.5% were female. There were 12,469 patients who received at least one statin prescription. Results from the reference case multivariable analysis indicated a reduced risk from statin exposure (HR: 0.85 (95% CI: 0.73–1.00) in COPD patients, but this result not statistically significant. Using the two recency-weighted modelling approaches, statin exposure was associated with a statistically significant reduction in lung cancer risk (recency-weighted cumulative dose, HR: 0.85 (95% CI: 0.77–0.93) and recency-weighted cumulative duration of use, HR: 0.97 (95% CI: 0.96–0.99). Multivariable analysis incorporating the negative control exposure was not statistically significant (HR: 0.89 (95% CI: 0.75–1.10).

**Conclusions:**

The results of this population-based analysis indicate that statin use in COPD patients may reduce the risk of lung cancer. While the effect was not statistically significantly across all exposure definitions, the overall results support the hypothesis that COPD patients might benefit from statin therapy.

## Introduction

Chronic obstructive pulmonary disease (COPD) is associated with considerable morbidity and mortality [1]. The disease is characterized by lung and systemic inflammation and is typically associated with several comorbidities [2, 3], including cardiovascular disease (CVD) [4]. HMG-CoA reductase inhibitors (statins) which are indicated for treatment of patients with established CVD [5], or at risk for CVD [6], are commonly used by COPD patients.

Evidence suggests that COPD patients are also at increased risk of lung cancer [7–10]. Multiple factors may increase COPD patients’ lung cancer risk, one of which is that COPD patients typically have a history of smoking [11]. However, it would appear that in a subset of patients, the increased risk of lung cancer extends beyond what can be attributed to their smoking status or history. In these patients, the additional risk of lung cancer may be due to increased lung and/or systemic inflammation that can increase their risk of cancer. Patients with concomitant CVD and COPD exhibit even higher levels of inflammation than COPD patients without CVD [12] and, as such, are likely at an even greater risk of lung cancer.

Statins have the potential to improve outcomes in COPD patients beyond their salutary effects on the cardiovascular system [13]. The objective of this analysis was to evaluate the association between statin use and the risk of lung cancer in a population-based cohort of COPD patients.

## Materials and methods

This study used population-based, linked, administrative data for the province of British Columbia (BC), Canada. These databases were: the Medical Services Plan (MSP) data file (physician billings within the universal health system) [14], the Discharge Abstract Database (DAD) (includes all hospital separations) [15], the PharmaNet datafile (contains all prescriptions dispensed in the province) [16], and the BC Cancer Registry file (details below) [17].

Classification as a COPD patient was based on having filled at least three prescriptions for an inhaled anticholinergic medication or a short-acting beta-agonist (SABA) in a one-year rolling time window, based on the PharmaNet datafile. Patients were required to be 50 years of age or greater. A subject’s index date was the date of the first of these prescriptions with follow-up period ending at the earliest of lung cancer diagnosis, death, or end of the study period. Details of the cohort are available in Table 1.

**Table 1:** Demographics of the cohort of COPD patients (*n* = 39,879)

Patient Characteristic	Value
Age	70.6 (SD: 11.2)
Age Distribution	
50 < 60	8356 (21.0%)
60 < 70	10,212 (25.7%)
70 < 80	12,436 (31.2%)
≥ 80	8785 (22.1%)
Female	21,273 (53.5%)
Income Quintile	
1	9701 (25.6%)
2	7864 (20.8%)
3	6854 (18.1%)
4	6381 (16.8%)
5	5696 (15.0%)
Health Authority	
Interior	8569 (21.6%)
Fraser	11,354 (28.6%)
Vancouver Coastal	7740 (19.5%)
Vancouver Island	7522 (18.6%)
Northern	2465 (6.2%)
Hospitalizations	6651 (16.7%
Any Reason	6651 (16.7%
COPD-related	1084 (2.7%)
CVD-related	512 (1.3%)
Charlson Comorbidity Category^a^	
0	31,354 (79.0%)
1	6303 (15.9%)
2	1176 (3.0%)
≥ 3	843 (2.1%)
Combination Therapy (ICS/LABA)	6585 (16.5%)
Physician Encounters (any reason)^b^	11 (3–22)
Number of Prescriptions Received (any reason)^b^	21 (7–44)

Values represent mean (standard deviation) or number (percentage) unless otherwise indicated

Where percentages do not add to 100% the reason is due to rounding

^a^Category 0 is a Charlson Score of 0, Category 1 is (0,2], Category 2 is (2, 3], Category 3 is > 3. This calculation excludes COPD, CVD, and cancer. ^b^Median and interquartile ranges

### Exposure measurement

Statin users were initially identified according to American Hospital Formulary Service (AHFS) codes [18]. While there were several different statins prescribed to the cohort of COPD patients, a class effect was assumed [19]. The period of exposure assessment started from the index date until the end of follow-up (see Supplementary Material). We used several different analytic approaches for defining medication exposure. Exposure status for all methods was time-depdendent; allowing for a patient’s exposure status to vary over the follow-up period can result in more statistical power to detect moderate effects, and minimizes the likelihood of biases, such as immortal time bias [20]. This analysis used three ‘conventional’ measures (i-iii) and two recency-weighted approaches (iv, v): (i) time-dependent statin exposure was the reference case analysis and classified those patients who had filled a statin prescription at any given time during the follow-up period (after the index date) as ‘exposed’, and those who did not as ‘unexposed’. To calculate cumulative years of use (ii) the days supplied of each prescription was aggregated during the follow-up period for each patient and divided by 365 to determine the total years of statin use. The cumulative dose (iii) was calculated using the dose of medication prescribed multiplied by the quantity prescribed. Statin prescriptions were converted to equivalent doses of atorvastatin (the most frequently dispensed statin in the cohort) [21]. The weighted cumulative duration of use (iv) method was employed to account for the duration of medication use while also accounting for when, during follow-up, the prescription was filled with respect to the outcome [22]. To account for when during follow-up the prescription was filled, a function was estimated that weighted prescriptions filled closer to the date of the event, or end of follow-up, greater than those that were filled earlier in the follow-up period, while also respecting the latency period (such that exposures that are within this period are not considered). The shape of the weighting function was based on previous literature employing this method [22–24]. This method assumes that while the duration of use is important, the exposures occurring immediately previous to the event, again respecting the latency period, confer a greater protective effect than exposures that occurred earlier in the follow-up period. Similarly, the recency-weighted cumulative dose (v) exposure definition was used to simultaneously account for both the cumulative dose and the time point at which the prescription was filled [22]. The same weighting function was employed which assigned a greater weight to prescriptions filled more proximal to the outcome, while respecting the latency period.

### Outcome ascertainment: lung cancer diagnosis

Lung cancer cases were retrieved the BC Cancer Registry file [17] which contains confirmed cancer diagnoses for the entire population of British Columbia, Canada. This database provides information on the date of diagnosis, cancer histology, and death date. This data file also provided information on each lung cancer case to classify lung cancers as: (i) non-small cell lung cancer (NSCLC); (ii) small cell lung cancer (SCLC); and (iii) ‘other’.

### Latency period

To attempt to appropriately classify statin exposure with respect to the pathogenesis of lung cancer, a one-year (365 day) latency period (or ‘lag’ period) was applied in the primary analysis [25]. That is, any dispensed statin prescription which was filled in the one-year period prior to lung cancer diagnosis, death, or censoring, was not considered as an exposure because lung cancer pathogenesis is likely have already been initiated during this period, and any exposure during this period was assumed to not influence lung cancer development. Each of the approaches to defining medication exposure described below incorporated this latency period, and the assumption of a one-year latency period was subjected to several sensitivity analyses.

### Adjustment for potential confounders

Potential confounders of the association between statin exposure and lung cancer diagnosis were incorporated into the multivariable model and assessed in the one-year period preceding the latency period. Demographic covariates included: age, sex, neighborhood income quintiles based neighborhood of residence, and the health authority (regional health service) where the patient resided. For each patient, the number of prescriptions dispensed (excluding statins), the number of hospital encounters, the number of inpatient hospital stays, and the number of physician encounters were calculated. Moreover, to account for comorbidities experienced by the patient during the follow-up period, the Charlson Comorbidity Index (CCI) was calculated based on health services records, excluding COPD, CVD, and cancer [26, 27].

### Statistical analysis

A Cox regression model was used to estimate the risk of lung cancer diagnosis based on statin exposure. To identify potential confounders to be included the multivariable model, a series of bivariate regression analyses were carried out to determine candidate covariates for the final multivariable model (see Table 2). Each covariate was added to the multivariable model via a stepwise selection comparing Akaike Information Criterion (AIC) values, with lower AIC values indicating a better model fit [28]. Hazard ratios and associated 95% confidence intervals are reported for each of the exposure metrics as: (a) bivariate analyses (Table 3); (b) multivariable age- and sex- adjusted analyses (Table 3); and (c) ‘fully’ adjusted multivariable analyses, with time to lung cancer diagnosis as the outcome (Table 4). Based on our previous research [29], an interaction term for statin and ICS use was also evaluated.

**Table 2:** Bivariate, and age/sex adjusted regression results (hazard ratios and 95% confidence intervals) for each exposure definition with time to lung cancer diagnosis as the outcome

Exposure Metrics	Bivariate			Age and Sex Adjusted		
	HR	95% CI LL	95% CI UL	HR	95% CI LL	95% CI UL
Time-dependent statin exposure	0.88	0.76	1.02	0.87	0.74	1.01
Cumulative Years of Use	0.97	0.91	1.02	0.96	0.91	1.02
Cumulative Dose^a^	0.99	0.98	1.00	0.99	0.98	1.00
Recency-Weighted Cumulative Duration of Use	0.87	0.79	0.95	0.86	0.79	0.94
Recency-Weighted Cumulative Dose	0.98	0.96	0.99	0.98	0.96	0.99

*HR* hazard ratio, *Ref* reference category, *CI* confidence interval, *LL* lower limit, *UL* upper limit

^a^Measured as a continuous variable (grams)

**Table 3:** Bivariate regression model results, with time to lung cancer diagnosis as the outcome, for covariates to be considered for inclusion in the multivariable model

Covariate	Hazard Ratio	95% Confidence Interval		***p***-value
Age	1.01	1.00	1.01	0.0011
Age Categories				
< 60	Ref	Ref	Ref	Ref
[60, 70)	2.02	1.67	2.43	<.0001
[70, 80)	2.33	1.95	2.80	<.0001
≥ 80	1.29	1.03	1.61	0.241
Sex (Male)	1.39	1.24	1.56	<.0001
Health Authority				
Interior	1.29	0.98	1.71	0.0744
Fraser	1.23	0.93	1.62	0.1485
Vancouver Coastal	1.04	0.78	1.40	0.7687
Vancouver Island	1.46	1.10	1.94	0.0084
Northern	Ref	Ref	Ref	Ref
Income Quintile				
5	Ref	Ref	Ref	Ref
4	1.27	1.03	1.57	0.0246
3	1.14	0.92	1.41	0.2152
2	1.23	1.00	1.50	0.0491
1	1.24	1.02	1.51	0.0305
Total Number of Prescriptions	1.00	0.99	1.00	<.0001
Charlson Comorbidity Score (Continuous)	1.06	0.97	1.16	0.1853
Charlson Comorbidity Score (Categorical)				
0	Ref	Ref	Ref	Ref
1	1.15	0.98	1.36	0.0931
2	0.94	0.62	1.40	0.7459
≥ 3	0.90	0.55	1.48	0.6814
Inpatient Stay	3.57	3.16	4.03	<.0001
Number of hospitalizations	1.66	1.64	1.68	<.0001
COPD-related hospitalization	2.56	2.00	3.27	<.0001
CVD-related hospitalization	1.04	0.58	1.88	0.8958
Combination Therapy (ICS/LABA)	1.27	1.11	1.47	0.007
Number of physician encounters	1.02	1.02	1.02	<.0001
Oral glucocorticoid use	1.09	0.91	1.30	0.3399

*HR* hazard ratio, *AIC* Akaike Information Criterion, *CI* confidence interval, *LL* lower limit, *UL* upper limit

**Table 4:** Multivariable regression results for each statin exposure metric with time to lung cancer diagnosis as the outcome variable

Exposure Metric	Multivariable Regression^a^				
	HR	95% CI LL	95%CI UL	*p*-value	AIC
Time-Dependent Statin Exposure	0.85	0.73	1.00	0.050	19,132
Cumulative Years of Use	0.95	0.90	1.01	0.118	19,133
Cumulative Dose^b^	0.99	0.98	1.00	0.128	19,133
Recency-Weighted Duration of Use	0.85	0.77	0.93	0.001	19,122
Recency-Weighted Cumulative Dose	0.97	0.96	0.99	0.002	19,124

*HR* hazard ratio, *AIC* Akaike Information Criterion, *CI* confidence interval, *LL* lower limit, *UL* upper limit

^a^Multivariable regression analysis was adjusted for the following covariates: age, sex, region, income quintile, inpatient hospitalization, number of physician encounters, COPD hospitalization, the year of cohort entry, Charlson Comorbidity Score, the total number of prescriptions received, oral glucocorticoid use, and time-dependent ICS exposure. ^b^Measured as a continuous variable (grams)

### Sensitivity analyses

The primary analysis assumed a one-year latency period for lung cancer. To test this assumption, the latency period was: (i) reduced to zero; (ii) reduced to 6 months; and (iii) extended to 2 years. Second, lung cancer incidence is low for patients less than 65 years of age [30, 31]. Therefore, a sensitivity analysis was conducted in which the cohort of COPD patients was restricted to 65 years and over to evaluate whether statin exposure resulted in a similar effect on lung cancer risk under this restriction. Using the same model developed for the main multivariable analysis, the lung cancer cases were restricted to either SCLC or NSCLC (as described above), and the association between statin use and these specific cancer types was evaluated and is presented in Table 2 below.

### Negative control exposure

An alternative medication class was identified for which there was no evidence of an association between this medication and the risk of developing cancer. This approach is similar to a placebo in a trial setting; the placebo, similar to the negative control exposure, should have no association with the study outcome [32]. Therefore, if an association exists between the negative control exposure, similar to what was found in the primary analysis, it is likely that the original result was due to confounding and provides evidence for the absence of a true association [32–34]. Calcium channel blockers (CCB) were chosen as the negative control exposure based on a review of the literature which showed no association between CCB and lung cancer risk, nor evidence supporting an association with cancer, generally [35–37]. Consistent with existing literature that has used negative control exposures, time-dependent CCB exposure was included in a bivariate Cox regression model, then in the fully-adjusted multivariable model [33, 38].

## Results

A cohort of 39,879 COPD patients was identified that met the study inclusion criteria. The mean age of the patients on their index date was 70.6 (SD: 11.2) years, 53.5% were females, and the mean follow-up time among patients in the COPD cohort was 5.1 years. There were 994 cases of lung cancer identified within the COPD cohort. Additional characteristics of the cohort of COPD patients are presented in Table 3.

### Statin use

There were 12,469 COPD patients who received at least one prescription for a statin during the study period, and a total of 258,458 statin prescriptions were dispensed. This resulted in an average of approximately 21 prescriptions per patient with atorvastatin (> 55%) being the most commonly prescribed.

### Bivariate results

In the bivariate analyses (Table 2), statin exposure was not significantly associated with lung cancer risk using any of the conventional methods (time-dependent exposure, cumulative years of use, cumulative dose), though the direction of the effect was in the a priori expected direction; that is, a reduction in the risk of lung cancer associated with statin exposure. The two recency-weighted exposure metrics, however, both showed a reduction in the risk of lung cancer by 13% per year of statin use (HR for recency-weighted duration of use: 0.87 (95% CI: 0.79–0.95) and 2% per gram of statin use (HR for recency-weighted cumulative dose: 0.98; 95% CI: 0.96–0.99).

### Multivariable analysis

In multivariable analysis, the hazard ratio for statin exposure was in the expected direction but was not statistically significant for the conventional exposure metrics ((i)-(iii) listed above). For these exposure metrics, the largest reduction of risk resulted from the reference case exposure definition, where time-dependent statin exposure was associated with a 15% decrease in lung cancer risk, but was not statistically significant (HR: 0.85 (95% CI: 0.73–1.00), at an absolute threshold for statistical significance of alpha equal to 0.05. When comparing AIC values, the model employing a time-dependent statin exposure metric provided the best fit among these metrics. Exposure classified based on the cumulative dose of statin received had the poorest AIC value, and the estimated HR was not statistically significant for lung cancer risk (HR: 0.99 (95% CI: 0.98–1.00)).

The two recency-weighted approaches had better (lower) AIC values compared to the conventional time-dependent exposure definitions, exhibiting approximately a ten-point difference. The estimated multivariable HR for the recency-weighted duration of use exposure metric showed a significant reduction in lung cancer risk from per year of statin use (HR: 0.85 (95% CI: 0.77–0.93) and the recency-weighted cumulative dose measure showed a 5% reduction in lung cancer risk per gram of statin (HR: 0.97 (95% CI: 0.96–0.99)). Of all the models incorporating time-dependent covariates in multivariable analysis, the recency-weighted duration of use exposure definition had the best AIC value (19122). Full results of multivariable analysis, along with AIC values, are presented in Table 4.

Our previous work has shown that inhaled corticosteroid (ICS) use is associated with a reduced risk of lung cancer [29]; therefore, an interaction term was added to the multivariable model to evaluate whether there is a synergistic effect between statin and ICS use in reducing the risk of lung cancer. However the interaction term was not statistically significant (HR: 1.01 (95% CI: 0.72–1.42)), therefore not lending to support to the idea of a synergistic effect of concurrent statin and ICS use. Addition of statin use to the multivariable model including ICS use did not alter the significant association of ICS, suggesting an independent protective effect.

### Lung cancer histology

Of the 994 cases of lung cancer identified within the COPD cohort, 854 were classified as non-small cell lung cancer (NSCLC) and 117 were classified as small cell lung cancer (SCLC). The distribution of these classifications aligned with estimates from other jurisdictions that reported approximately 15% of lung cancer cases are classified as SCLC [31, 39]. The estimated hazard ratios for the association between statin use the development of NSCLC was 0.83 (95% CI: 0.70–0.99) for time-dependent exposure and 0.83 (95% CI: 0.75–0.92) for the recency-weighted duration of use metric. For SCLC, the estimated HRs for each metric of statin exposure were not statistically significant (Table 5), however, this result may have been due to the lower number of SCLC cases observed (*n* = 117).

**Table 5:** Evaluation of association between statin exposure and lung cancer histology

	Multivariable Regression			
	HR	95% CI LL	95%CI UL	***p*** value
NSCLC				
Time-Dependent Statin Exposure^a^	0.83	0.70	0.99	0.0349
Recency-Weighted Duration of Use^b^	0.83	0.75	0.92	0.0004
SCLC				
Time-Dependent Statin Exposure	1.18	0.77	1.80	0.4542
Recency-Weighted Duration of Use	1.04	0.82	1.32	0.7561

*HR* hazard ratio, *CI* confidence interval, *LL* lower limit, *UL* upper limit, *NSCLC* non-small cell lung cancer, *SCLC* small cell lung cancer. ^a^ This is the reference-case for the analysis. ^b^ The recency weighted duration of use exposure metric is presented because it was selected as the best model based on AIC values (an a priori criterion)

### Sensitivity analyses

Several sensitivity analyses were performed to explore how different specifications of the latency period might affect the results of this study. These results are presented in Table 6. When the latency period was eliminated altogether, statin use was not significantly associated with lung cancer risk using a time-dependent statin exposure metric. When a six-month latency period was applied, the estimated HR for each exposure metric was not statistically significant but, again, was in the expected direction. When the latency period was extended to two years, the association between lung cancer risk and statin use was statistically significant, suggesting an almost 40% reduction in lung cancer risk from statin use compared to non-use (HR: 0.62 (95% CI: 0.52–0.73). For the recency-weighted duration of use exposure metric, the results were similar under this assumption, where the estimated multivariable HR suggested a greater than 40% reduction in lung cancer risk conferred from statin use (HR: 0.57 (95% CI: 0.49–0.66).

**Table 6:** Sensitivity analyses: evaluation of different lengths of the latency period and a cohort age restriction, using time-dependent exposure and the recency-weighted duration of exposure metrics, with time to lung cancer as the outcome

	Multivariable Regression			
	HR	95% CI LL	95%CI UL	***p***-value
Latency Period				
None				
Time-Dependent Statin Exposure^a^	1.00	0.87	1.16	0.9698
Recency-Weighted Duration of Use^b^	1.04	0.97	1.12	0.2754
6 months				
Time-Dependent Statin Exposure	0.96	0.82	1.12	0.5808
Recency-Weighted Duration of Use	1.00	0.92	1.08	0.9214
1 year^c^				
Time-Dependent Statin Exposure	0.85	0.73	1.00	0.05
Recency-Weighted Duration of Use	0.85	0.77	0.93	0.0006
2 years				
Time-Dependent Statin Exposure	0.62	0.52	0.73	< 0.0001
Recency-Weighted Duration of Use	0.57	0.49	0.66	< 0.0001
Cohort (Age ≥ 65 years)				
Time-Dependent Statin Exposure	0.72	0.60	0.86	0.0004
Recency-Weighted Duration of Use	0.78	0.70	0.87	< 0.0001

^a^This is the reference-case for the analysis. ^b^The recency-weighted duration of use exposure metric is presented because it was selected as the best model based on AIC values. ^c^A one-year latency period was assumed in the primary analysis and is presented here for comparison purposes

In another analysis, the cohort of COPD patients was then restricted to those 65 years of age and over to reflect the fact the lung cancer typically occurs in patients near this age. The estimated HR for time-dependent statin exposure, under the reference latency period, was 0.72 (95% CI: 0.60–0.86) showing a protective effect from statin use on lung cancer risk. Similarly, using the recency-weighted duration of use exposure metric, statin use was associated with an 22% decrease in lung cancer risk per year of statin use (HR: 0.78 (95% CI: 0.70–0.87).

An additional analysis was conducted, using a negative control exposure, to detect whether the results of the primary analysis were inherently biased or confounded. To do so, the association between time-dependent CCB exposure and lung cancer risk was explored in a multivariable model. In the multivariable (without statin and ICS exposure), and in multivariable analysis including time-dependent ICS and statin exposure, there was no association between the CCB exposure (the negative control exposure) and lung cancer using any definition of medication exposure (*i.e.*, time-dependent CCB exposure HR was 0.89 (95% CI: 0.75–1.10) and 0.92 (95% CI: 0.77–1.11) when included in the multivariable model with the statin and ICS exposure variables).

## Discussion

This study evaluated the association between lung cancer risk and statin exposure in a population-based cohort of COPD patients using an array of metrics for quantifying medication exposure. While statin exposure was not statistically significantly associated with a reduction in lung cancer risk across all exposure definitions, the overall results of this study do suggest that statin use in COPD patients reduces the risk of lung cancer and lends support to the hypothesis that patients with COPD might benefit from statin therapy [13]. Beyond superior AIC values, the advantage of the recency-weighted approach is intuitive; it implies that the duration of statin use is important, but also when that use occurs, with respect to the outcome, is also important. The results of this analysis also suggest that statin use may reduce the risk of lung cancer in COPD patients aged sixty-five or greater, and that the protective effect of statins might be greater for NSCLC.

The results of this analysis are strengthened by a plausible biological mechanism by which statin use might reduce lung cancer risk. Lung and systemic inflammation, which may result from COPD or, indeed, may be a cause of COPD [40], is associated with increased lung cancer risk. Evidence suggests that elevated levels of markers for systemic inflammation are associated with a one to three times greater likelihood of lung cancer, independent of smoking status. Previously conducted studies have reported that statin use also appears to be associated with reduced levels of systemic inflammation. Several trials conducted which demonstrated that statin use is associated with reduced levels of systemic inflammation For example, in the JUPITER trial, statin use was associated with a 37.4% reduction in systemic inflammation after 48 months relative to placebo [41]. In addition to their effect on systemic inflammation, there is recently published evidence from pilot studies that suggest statin use may actually reduce markers for local/pulmonary inflammation [42, 43] which has also been associated with increased lung cancer risk.

While studies have evaluated statin use and cancer risk [44, 45], only a limited number of studies have specifically evaluated statin use and lung cancer risk. Marelli et al. [46] conducted an analysis using electronic medical records of 45,857 matched pairs of adult Americans with an average of 4.6 years of follow-up, and found no statistically significant relationship between exposure to statins and risk of any cancer compared with no statin exposure (HR: 1.04 (95% CI: 0.99–1.09)). Bonovas et al. [47] evaluated the association between statin use and cancer risk using seven large randomized controlled trials (RCTs). The results of their study showed no significant association between statin use and the risk of any cancer; meta-regression analysis showed some evidence that statin use may decrease cancer incidence in younger patients [47]. Setoguchi et al. [48] focused on an elderly population and found no statistically significant reduction in lung cancer incidence associated with statin use. Conversely, a meta-analysis of case-control studies produced an overall pooled OR of 0.71 (95% CI: 0.56–0.89) for any cancer and a non-significant result of 0.75 (95% CI: 0.50–1.11) specifically for lung cancer development [49]. Khurana et al. [50] focused on statin use prior to lung cancer diagnosis in the United States and reported a significant protective effect for statin (OR: 0.55 (95% CI: 0.52–0.59)). The same study found that the duration of statin use also impacted the likelihood of lung cancer development; patients that had used a statin for greater than six months had an OR of 0.45 (95% CI: 0.42–0.48). The results were similar for smokers who used statins for greater than six months with an OR of 0.47 (95% CI: 0.43–0.51) compared to patients with no statin use.

While the evidence is far from unanimous, previously completed observational studies support the idea that statins might reduce cancer risk [51] but only one study previously found statistically significant reductions in lung cancer risk, specifically associated with statin use [50]. The results of this study, however, are not generalizable; the analysis used data from the United States Veterans Health Administration which was almost exclusively male (97.9%). Evidence also suggests statins have been shown to improve survival for those who continued statin therapy after cancer diagnosis [52]. Therefore, this study provides an important contribution to the evidence for statin use and lung cancer risk.

In addition to the results of this study showing a potential protective effect of statin use and a reduction in lung cancer risk, the results also suggested that statin use reduced the risk of NSCLC, specifically. The results for SCLC, however, were not statistically significant. The absence of a differential effect is supported by the work of Chaturvedi et al. [53] which found that elevated levels of systemic inflammation were associated with lung cancer, generally, and there was no statistically significant difference in the levels of systemic inflammation were elevated in patients that developed SCLC or NSCLC. That is, systemic inflammation appeared to be significantly associated with both types of lung cancer. Therefore, if the mechanism by which statins are thought to reduce the risk of lung cancer in COPD patients is via a reduction in systemic inflammation, and elevated levels of systemic inflammation are associated with both SCLC and NSCLC, the results of the analysis suggesting statins reduce the risk of NSCLC are consistent. While the results of the analysis with SCLC and statin use were not statically significant, this is likely due to the low number of observed SCLC cases.

Previous studies have reported that statin use in COPD patients is associated with a slowing in the decline in lung function [54], and a reduction in the risk of all-cause mortality [13]. This evidence suggests that statins have pleiotropic effects in COPD patients and should be considered as a potential therapy beyond hypercholesterolemia [55]. This evidence might also suggest that identification of patients with elevated levels of systemic inflammation might offer prognostic information and allow for targeted statin treatment.

The study has several strengths. First, it uses population-based administrative data for an entire Canadian province which significantly enhances the generalizability of its findings compared to other existing studies. Moreover, it was possible to link this administrative data to high-quality registry data from the British Columbia Cancer Registry file to accurately identify the diagnosis date and histology of lung cancer. As such, the data used in this study provides the highest level of real-world effectiveness evidence. Moreover, where previous studies may have lacked adequate power to identify an association between statin exposure and lung cancer risk, this study did not. Second, it is the first study that has used an extensive list of medication exposure definitions to address the question of whether statins might confer benefit, in terms of reduced lung cancer risk, in COPD patients. Statin exposure was statistically significant in several adjusted analyses and for all exposure definitions when the cohort was restricted to patients aged 65 and over, which enhances the robustness of these results. In addition, the use of recency-weighted approaches, which showed superior model fit to the conventional exposure definitions, is also a key strength, and may provide a useful methodological approach in future studies evaluating cancer risk associated with medication use. This method of defining medication exposure implies that the duration of statin use is important, but also that when the use occurs, proximal to the outcome, is important. Third, the incorporation of a latency period associated with lung cancer, and, therefore, not classifying medication exposures immediately preceding lung cancer diagnosis as relevant exposures, is a strength of this study and should inform future observational studies in cancer research. Finally, the use of a negative control (in this case, CCB exposure) to detect if the results of the analysis were due to bias or confounding is a major strength of this study, which provides support for a true association between statin exposure and lung cancer risk, and significantly enhances the robustness of the results.

There are several limitations to this study which require acknowledgement. First, the administrative data used in this study did not include any clinical variables that would be useful in the analysis (for example, level of systemic inflammation or lung function). As such, COPD patients were not identified according to spirometry but rather based on their individual prescription records. However, previous studies have used a similar method for identifying COPD patients [56, 57] and this is believed to be a sensitive approach to identifying these patients. It is possible that this approach identified some patients as having COPD, when, in reality, they did not. To attempt to provide a more specific definition of COPD, we also added the requirement for a physician encounter with an ICD-9 code (491, 492, and 496) consistent with COPD within one year of the index date. This reduced the size of our cohort by less than approximately 10% and is an approach is similar that used by Curkendall et al. [58] and Gershon et al. [59]. Using a more restrictive approach, for example by requiring a hospitalization for COPD, may have only identified patients with more severe disease. Therefore, this approach should enhance the generalizability of the study results. In addition, the focus of this research was not to study patients with COPD, but rather to evaluate lung cancer risk in patients at high risk for developing the disease. As such, while we attempted to identify a cohort of COPD patients, it is less important that patients have COPD, and more important that patients were at an increased risk of lung cancer in the cohort. It should also be noted that in BC, receipt of inhaled anticholinergic medication is indicative of a COPD diagnosis. Short-acting anticholinergic medications are only reimbursable if the patient has a clinical diagnosis of COPD and long-acting anticholinergic medications are reimbursable if patients have a post-bronchodilator spirometric FEV1/FVC < 0.7 and FEV1 < 65% of predicted, and remained persistently symptomatic on at least three months of continuous short-acting anticholinergic therapy. Second, smoking status and smoking history were also not captured in our administrative data. However, previous literature does suggest that the majority of the cohort will have a history of smoking so it would be expected that the majority of this cohort did, indeed, have a history of smoking. For example, estimates suggest that approximately 85% of COPD patients have a history of smoking [60–63]; therefore, we could crudely assume that approximately 34,000 patients identified in the cohort of COPD patients had a history of smoking. It might also be the case that smokers may have more severe COPD, and that this group of patients would be more likely to develop lung cancer, which would conservatively bias the results for the effect of statins. Third, while a strong effect was observed for statin use and lung cancer risk, this study is subject to the limitations of all observational studies whereby we cannot be certain that there is an element of unmeasured or residual confounding that explains the study results. In order to mitigate this possibility, a systematic approach to identifying potential confounders to be included in the multivariable analysis was adopted. The magnitude of the effect size also reduces the likelihood that the protective effect of statin use could be explained by residual confounding. Moreover, the use of a one-year latency period reduces the likelihood that the study results could be explained by protopathic bias. Finally, the study results were also consistent for a variety of medication exposure definitions and in several sensitivity analyses.

## Conclusions

This analysis demonstrated that statin use in COPD patients may reduce the risk of lung cancer. Using the recency-weighted approaches to capture statin exposure resulted in statistically significant hazard ratios, and these two models were deemed superior based on an a priori specified criterion. In sub-group analyses, statin exposure was significantly associated with a reduction in lung cancer risk for patients 65 years of age or greater, across all exposure metrics, and also for NSCLC. These results were further strengthened by an analysis incorporating a negative control exposure to detect residual confounding or bias. These results strengthen the hypothesis that there may be a segment of COPD patients, likely characterized by elevated levels of inflammation, that could benefit substantially from statin therapy.

## Supplementary information

**Additional file 1: Figure S1.** A graphical representation of the latency period, and how medication exposure is considered with respect to the latency period.

## Data Availability

The datasets generated and/or analyzed during the current study are not publicly available due to privacy restrictions. Descriptions of each of the databases are available from Population Data BC (British Columbia, Canada) (https://www.popdata.bc.ca/).

## Abbreviations

AIC: Akaike Information Criterion
AHFS: American Hospital Formulary Service
BC: British Columbia
CCB: Calcium channel blockers
CVD: Cardiovascular disease
COPD: Chronic obstructive pulmonary disease
CI: Confidence interval
DAD: Discharge abstract database
FEV1: Forced expiratory volume in 1 s
FVC: Forced vital capacity
HR: Hazard ratio
ICS: Inhaled corticosteroid
MSP: Medical Services Plan
NSCLC: Non-small cell lung cancer
OR: Odds ratio
SABA: Short-acting beta-agonist
SCLC: Small cell lung cancer
