# Supplementary material for: Statin use and lung cancer risk in chronic obstructive pulmonary disease patients: a population-based cohort study
Source: Respir Res. 2020 May 19;21:118. doi: 10.1186/s12931-020-01344-w (PMC7236956; doi:10.1186/s12931-020-01344-w)
Supplement: Supplementary file 1 — Additional file 1: Figure S1. A graphical representation of the latency period, and how medication exposure is considered with respect to the latency period. [file 12931_2020_1344_MOESM1_ESM.docx]

**Supplementary Material**

Figure S1. A graphical representation of the latency period, and how medication exposure is considered with respect to the latency period.
